# PMCA4 inhibition does not affect cardiac remodelling following myocardial infarction, but may reduce susceptibility to arrhythmia

**DOI:** 10.1038/s41598-021-81170-2

**Published:** 2021-01-15

**Authors:** Nicholas Stafford, Min Zi, Florence Baudoin, Tamer M. A. Mohamed, Sukhpal Prehar, Daria De Giorgio, Elizabeth J. Cartwright, Roberto Latini, Ludwig Neyses, Delvac Oceandy

**Affiliations:** 1 Division of Cardiovascular Sciences, Faculty of Biology, Medicine and Health, Manchester Academic Health Science Centre, The University of Manchester, Manchester, UK; 2 Department of Medicine, Institute of Molecular Cardiology, University of Louisville, Louisville, KY USA; 3 Faculty of Pharmacy, Zagazig University, Zagazig, Egypt; 4 Department of Cardiovascular Medicine, Mario Negri Institute for Pharmacological Research, Milan, Italy; 5 Simply Uni, Sète, France

**Keywords:** Cardiovascular biology, Cardiac hypertrophy, Cardiovascular diseases

## Abstract

Ischaemic heart disease is the world’s leading cause of mortality. Survival rates from acute myocardial infarction (MI) have improved in recent years; however, this has led to an increase in the prevalence of heart failure (HF) due to chronic remodelling of the infarcted myocardium, for which treatment options remain poor. We have previously shown that inhibition of isoform 4 of the plasma membrane calcium ATPase (PMCA4) prevents chronic remodelling and HF development during pressure overload, through fibroblast mediated Wnt signalling modulation. Given that Wnt signalling also plays a prominent role during remodelling of the infarcted heart, this study investigated the effect of genetic and functional loss of PMCA4 on cardiac outcomes following MI. Neither genetic deletion nor pharmacological inhibition of PMCA4 affected chronic remodelling of the post-MI myocardium. This was the case when PMCA4 was deleted globally, or specifically from cardiomyocytes or fibroblasts. PMCA4-ablated hearts were however less prone to acute arrhythmic events, which may offer a slight survival benefit. Overall, this study demonstrates that PMCA4 inhibition does not affect chronic outcomes following MI.

## Introduction

Cardiovascular diseases (CVD) account for 45% of all mortality across Europe^1^, and are responsible for more lives in the US than all cancer and chronic lung diseases combined^2^. Of these, ischaemic heart disease accounts for nearly half of all deaths due to CVD^3^, and is the leading cause of death worldwide^4^. Whilst mortality from acute myocardial infarction (MI) has fallen in recent decades in the developed world, thanks in large part to the rise in primary percutaneous coronary interventions^5^, this has led to a concomitant rise in the prevalence of heart failure (HF) as a result of chronic post-infarct left ventricular remodelling^6^.

Despite the significant improvements in survival from infarction, only a handful of new pharmacological therapies aimed at preventing and treating heart failure have been approved in the past two decades^7^. During this time, large numbers of seemingly promising approaches, such as stem cell and gene-based therapies, have failed to significantly reduce mortality or improve cardiac function upon reaching clinical trials^8,9^. Consequently, frontline prevention and management of HF remains centred around targeting the renin–angiotensin–aldosterone system and sympathetic nervous system to delay disease progression^10^, rather than intrinsic cardiac factors which regulate the remodelling process.

One promising target for cardiac remodelling that has emerged in recent years is the calcium signalling protein plasma membrane calcium ATPase isoform 4 (PMCA4). PMCA4 is a ubiquitously expressed pump which extrudes calcium from the cytosol to the extracellular space, and in non-excitable cells it can act as a major regulator of intracellular calcium concentration^11^. In cardiomyocytes we and others have shown PMCA4′s contribution towards global calcium extrusion is minimal during excitation–contraction coupling^12,13^; however, through the control of local calcium and protein–protein interactions, it plays a key role in a regulating a number of pathways such as nitric oxide^14,15^, calcineurin-NFAT^16^ and tumour suppressor RASSF1-Erk^17^ signalling. Importantly, these roles have been shown to have functional implications in PMCA4 ablated or overexpressing mice, including the regulation of cardiac contractility^12,18,19^, hypertrophy^13,18,20^, blood pressure^21^, angiogenesis^22^ and sperm motility^23^.

Through the development of a specific inhibitor^24^, we have shown PMCA4 to be a druggable candidate in a number of cardiovascular settings including the lowering of blood pressure^25^, promotion of angiogenesis^26^, the treatment of cardiac hypertrophy and the prevention of HF^20^. Our previous study found that PMCA4 inhibition reversed cardiac remodelling in mouse hearts subjected to pressure overload, through a paracrine mechanism involving the secretion of the Wnt inhibitor sFRP2 from cardiac fibroblasts^20^. Given that Wnt activation also contributes towards remodelling and HF development following MI^27^, and that sFRP2 treatment has produced beneficial outcomes in the infarcted rat heart^28^, we conducted a study into the effects of PMCA4 inhibition after MI. Surprisingly, we found that PMCA4 inhibition did not affect chronic outcomes after MI, and therefore does not present a new target to prevent HF development in the infarcted heart.

## Results

### Cardiac structure and function are not improved in PMCA4^−/−^ mice after MI

**Figure 1:**
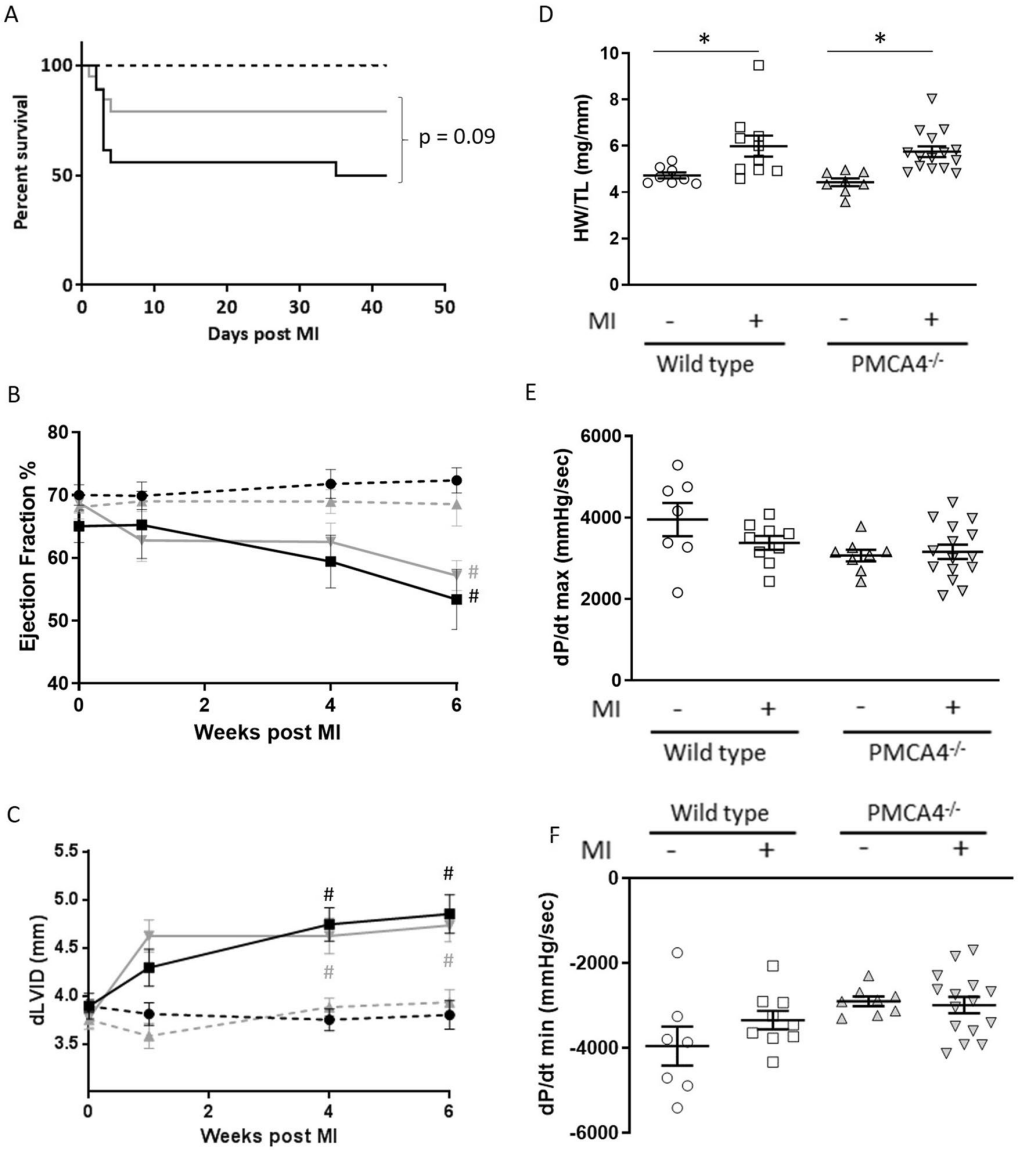
PMCA 4 global deletion does not affect cardiac structure or function post-MI. (**A**) Kaplan–Meier survival plots for wild type (black) and PMCA4^−/−^ mice (grey) followed for 6 weeks post MI (solid lines) or sham (dashed lines) surgery. Starting populations; WT sham n = 8, WT MI n = 18, PMCA4^−/−^ sham n = 8, PMCA4^−/−^ MI n = 19. (**B**) Ejection fraction and (**C**) Diastolic left ventricular diameter tracked for 6 weeks post-MI or sham surgery by echocardiography. # *p* < 0.05 versus sham control at matched time point (**D**) Heart weight/tibia length ratio 6 weeks post-MI or sham surgery. (**E**) dP/dt max and (**F**) dP/dt min 6 weeks post MI or sham surgery. WT sham n = 8, WT MI n = 9, PMCA4^−/−^ sham n = 8, PMCA4^−/−^ MI n = 15 * *p* < 0.05.

To assess the effects of *Pmca4* genetic ablation following myocardial infarction (MI), we performed surgery to permanently ligate the left anterior descending coronary artery in our global PMCA4 knockout mice (*PMCA4*^*−/−*^ mice). During 6 weeks follow-up, *PMCA4*^*−/−*^ mice experienced a 79% survival rate post-MI, compared to 50% survival in wild type (WT) littermate controls (Fig. 1A). Although there was a trend towards a higher survival rate in *PMCA4*^*−/−*^ mice, the difference did not reach statistical significance (*p* = 0.09). Examining the data further by gender, the survival benefit appeared restricted to female *PMCA4*^*−/−*^ mice, which showed 100% survival versus 50% in WT (*p* < 0.05—Supplementary Table S1). There was no difference by genotype in survival rates following MI in male mice. No deaths were observed over the 6 week period in mice of either genotype undergoing a sham operation, in which the chest was opened and heart exposed without ligation. In both MI groups the majority of deaths occurred acutely over the first 4 days following surgery. Only one instance of death from chronic cardiac failure was observed outside of this time period, at day 35 in the WT MI group.

Ejection fraction as measured by serial echocardiography at 1, 4 and 6 weeks post-MI did not differ between *PMCA4*^*−/−*^ and WT mice (Fig. 1B), although both genotypes experienced a decline in ejection fraction at 6 weeks post-MI compared to their respective sham controls. Ejection fraction declined comparably amongst sex-matched *PMCA4*^*−/−*^ and WT mice (Supplementary Table S1). Similarly, whilst MI led to significant cardiac dilation as measured by internal left ventricular diastolic diameter after 4 weeks in both genotypes, the extent was comparable amongst WT and *PMCA4*^*−/−*^ MI groups (Fig. 1C).

At the end of the 6 week follow-up period, haemodynamic analysis was performed by left ventricular catheterisation and tissues harvested for analysis. Normalised heart weight was increased in both MI groups compared to sham controls, but did not differ by genotype in either males or females (Fig. 1D and Supplementary Table S1). Furthermore, cardiomyocyte cross sectional area was similar in both the infarct border and remote regions of the myocardium amongst MI groups (Suppl. Fig. S1), indicating that the hypertrophic response to MI was not affected by *Pmca4* gene ablation. Haemodynamic analysis of cardiac contractility and lusitropy revealed no significant differences amongst groups either in sham or MI mice, as measured by the maximum and minimum rates of pressure change (dP/dt max and min, Fig. 1E,F). Taken together these results indicate that global *Pmca4* gene ablation may offer a slight but non-significant survival benefit during the acute phase following MI, but does not impact long term cardiac function or structure.

### Chronic cardiac remodelling is not affected by Pmca4 gene ablation

**Figure 2:**
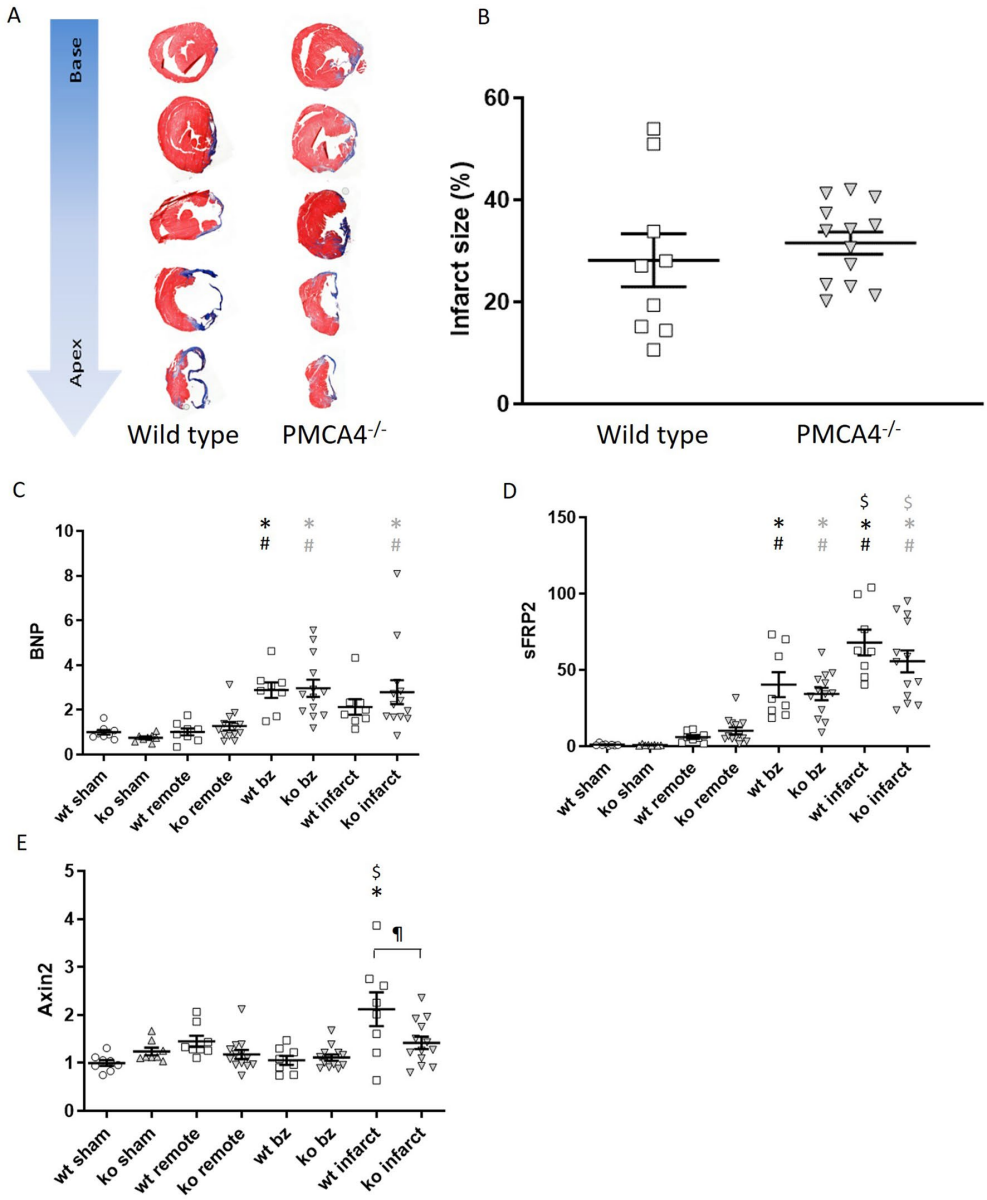
PMCA 4 global deletion does not affect chronic infarct size, remodelling or Wnt signalling post MI. (**A**) Representative masson’s trichrome stained sections from wildtype and PMCA4^−/−^ hearts following 6 weeks MI. Images were acquired using a 3D Histech Panoramic slide scanner and CaseViewer software version 2.4.0. (**B**) Quantification of infarct size measurements 6 weeks post-MI. WT n = 9, PMCA4^−/−^ n = 13. Regional gene expression was examined in WT (clear points) and PMCA4^−/−^ (grey points) hearts 6 weeks post sham or MI surgery for (**C**) Hypertrophic marker BNP (**D**) Wnt inhibitor sFRP2 and (**E**) Wnt transcriptional marker Axin2. bz = infarct border zone. Shams n = 8, WT MI n = 8, PMCA4^−/−^ MI n = 13. *p < 0.05 versus sham, # *p* < 0.05 versus remote, $ *p* < 0.05 versus border zone, ¶ *p* < 0.05 WT v KO infarct.

To confirm the in vivo structural and functional findings that *Pmca4* gene deletion did not alter the chronic response to MI, hearts were processed at the end of the 6 week period for histological and molecular analysis. Hearts were sectioned at 5 levels from base to apex and stained with Masson’s trichrome in order to assess the infarct size, as a percentage of the left ventricle (Fig. 2A). Infarct size was found to be comparable between *PMCA4*^*−/−*^ and WT mice (Fig. 2B) and did not differ by sex (Supplementary Table S1), suggesting that cardiomyocyte survival was not affected after MI.

In order to confirm that cardiac remodelling was not affected by *Pmca4* ablation, RNA was extracted from infarcted regions, as well as infarct border and remote regions of the left ventricle and from sham controls. qPCR analysis revealed that expression of brain natriuretic peptide (*Bnp*), a common marker for pathological cardiac remodelling, was increased in MI infarct and border regions compared to remote regions and sham hearts in both genotpyes (Fig. 2C); however, when comparing *Bnp* expression in each respective region of *PMCA4*^*−/−*^ and WT MI hearts no differences were observed. These results support the in vivo data showing that chronic cardiac remodelling was not altered in *PMCA4*^*−/−*^ mice after MI.

We have previously shown that beneficial outcomes in *PMCA4*^*−/−*^ mice during pressure overload are caused by a reduction in canonical *Wnt* signalling via increased levels of secreted frizzled related protein 2 *(sFRP2)*^20^. Therefore, to investigate whether a similar signalling mechanism was active following MI we also examined regional expression of *sFRP2* and *Wnt*/β-catenin target gene *Axin2*. In general *sFRP2* levels were higher in border and infarcted regions; however matched regional *sFRP2* expression was comparable in *PMCA4*^*−/−*^ and WT hearts (Fig. 2D). *Axin2* expression did not differ in sham, remote or border regions of knockout or WT hearts. Interestingly however, expression of the β-catenin target gene was higher in the infarcted myocardium of WT hearts compared to *PMCA4*^*−/−*^ infarcts*,* and also raised compared to sham and border regions (Fig. 2E). This may suggest some reduction in *Wnt* signalling in the infarct of *PMCA4*^*−/−*^ hearts, but overall the data indicates that *Pmca4* gene deletion does not affect chronic cardiac remodelling post-MI.

### PMCA4^−/−^ mice are less prone to acute arrhythmic events after MI

While we didn’t find *Pmca4* deletion to affect long term remodelling after MI, there did appear to be some survival benefit during the acute phase after MI (Fig. 1A). While one instance of sudden death occurred on each of the first four days post-MI in the knockout group (a total of 4 out of 19 mice), a substantial number of deaths occurred at day 2 or 3 post-MI in the WT control group (7 out of 18 mice). We aimed to investigate the potential cause of this by looking at the cardiac phenotype over the first 2 days post-MI, at a time point prior to the occurrence of sudden death in WT MI mice. 2 day survival rates were similar amongst *PMCA4*^*−/−*^ and control mice in this cohort, and did not differ when analysing separately by sex (Supplementary Table S2).

**Figure 3:**
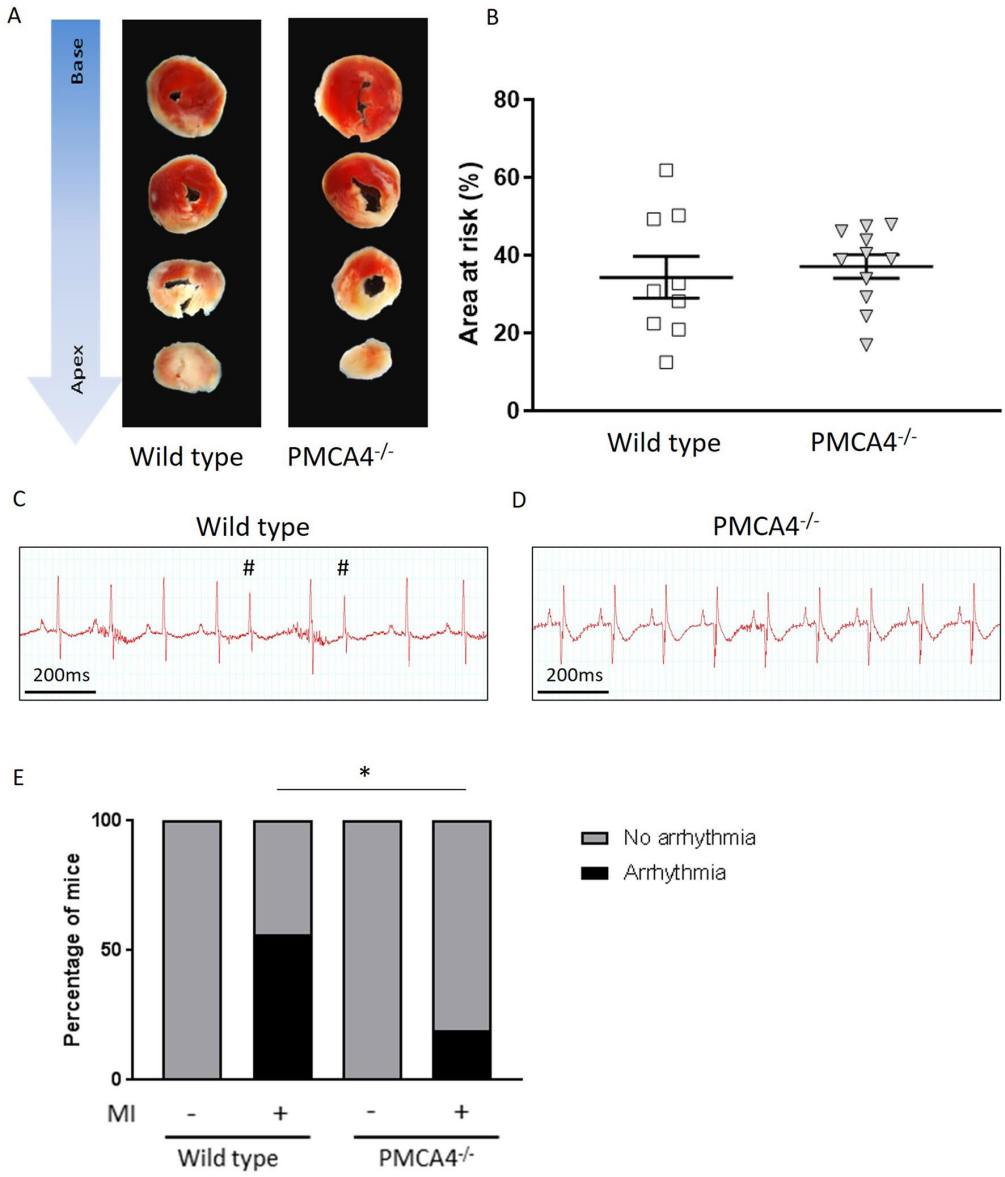
PMCA 4 global deletion may protect against acute arrhythmic events post-MI. (**A**) Representative TTC stained sections from wildtype and PMCA4^−/−^ hearts 2 days post MI. Images were acquired using a Leica S6D microscope with Leica MC170 HD camera, and Leica application suite EZ software. (**B**) Quantification of area at risk 2 days post-MI. WT n = 9, PMCA4^−/−^ n = 11. (**C**) Representative 3 lead ECG traces from wild type and (**D**) PMCA4^−/−^ mice day 1 post-MI. Premature contractions are indicated by #. (**E**) Percentage of mice displaying arrhythmia (black segments) upon ECG examination day 1 post MI. WT sham n = 8, WT MI n = 18, PMCA4^−/−^ sham n = 8, PMCA4^−/−^ MI n = 16 * *p* < 0.05.

We then analysed the area at risk through triphenyltetrazolium chloride (TTC) staining of heart sections 2 days after MI (Fig. 3A). Similar to the results obtained during 6 week infarct size analysis, we found no difference in area at risk between *PMCA4*^*−/−*^ and WT hearts at 2 days in either male or female mice (Fig. 3B and Supplementary Table S2), indicating that the extent of injury was comparable between groups and likely not the cause of any survival benefit.

The most common causes of death in the first days after LAD ligation are due to fatal arrhythmias or cardiac rupture^29,30^. We therefore performed 3 lead ECG analysis on the first day after MI surgery, prior to the occurrence of sudden death in WT mice. Traces were recorded for a period of 15 min in mice maintained under general anaesthesia, and assessed for the presence of arrhythmic beats. All sham mice were found to have a regular sinus rhythm throughout the period of recording. In contrast, 56% of WT MI traces exhibited ectopic beats during the 15 min period, significantly higher than the rate of 19% observed in *PMCA4*^*−/−*^ traces after MI (Fig. 3C–E). Arrhythmic events were higher in both male and female WT mice compared to those with *Pmca4* ablation (Supplementary Table S2). The premature complexes were narrow and not preceded by visible P waves, suggesting that they were atrial or junctional in origin (Fig. 3C). The rates of prevalence of arrhythmia closely match the overall rate of sudden death for each genotype, and could be associated with improved survival in *PMCA4*^*−/−*^ mice.

### Pmca4 deletion does not affect myocardial inflammation or fibrosis post-MI

**Figure 4:**
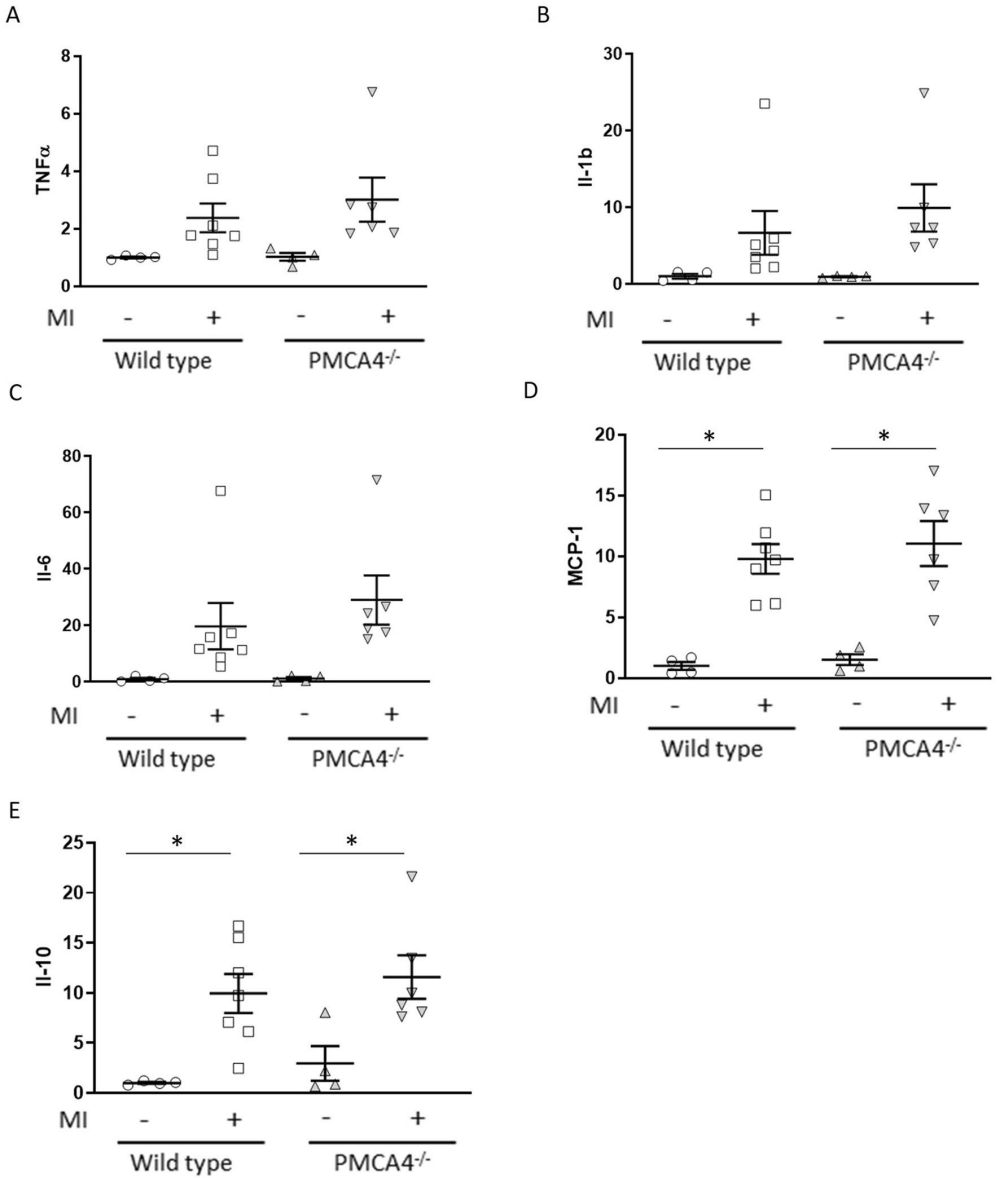
PMCA 4 global deletion does not affect the acute inflammatory response post-MI. Myocardial gene expression was examined by qRT-PCR in WT (clear points) and PMCA4^−/−^ (grey points) hearts 2 days post sham or MI surgery for pro-inflammatory mediators (**A**) TNFα, (**B**) Interleukin-1β, (**C**) Interleukin-6 and (**D**) MCP-1, in addition to anti-inflammatory cytokine (**E**) Il-10 Shams n = 4, WT MI n = 7, PMCA4^−/−^ MI n = 6. *p < 0.05.

Inflammation and fibrosis are major determinants of outcomes following myocardial infarction, and can both contribute towards the development of arrhythmia^29^. To examine whether these processes were affected in *PMCA4*^*−/−*^ mice we examined mRNA expression of inflammatory and fibrotic markers in heart tissue during the acute (2 day) and chronic (6 week) stages post-MI. Expression of pro-inflammatory mediators tumour necrosis factor-α (TNFα), interleukin-1β (Il-1β), Il-6 and monocyte chemoattractant protein-1 (MCP-1) was increased in MI compared to sham hearts 2 days after surgery, but did not differ between WT and *PMCA4*^*−/−*^ hearts (Fig. 4A–D). Likewise, levels of the anti-inflammatory cytokine Il-10 were elevated in MI hearts at this acute stage, but similar amongst genotypes (Fig. 4E).

**Figure 5:**
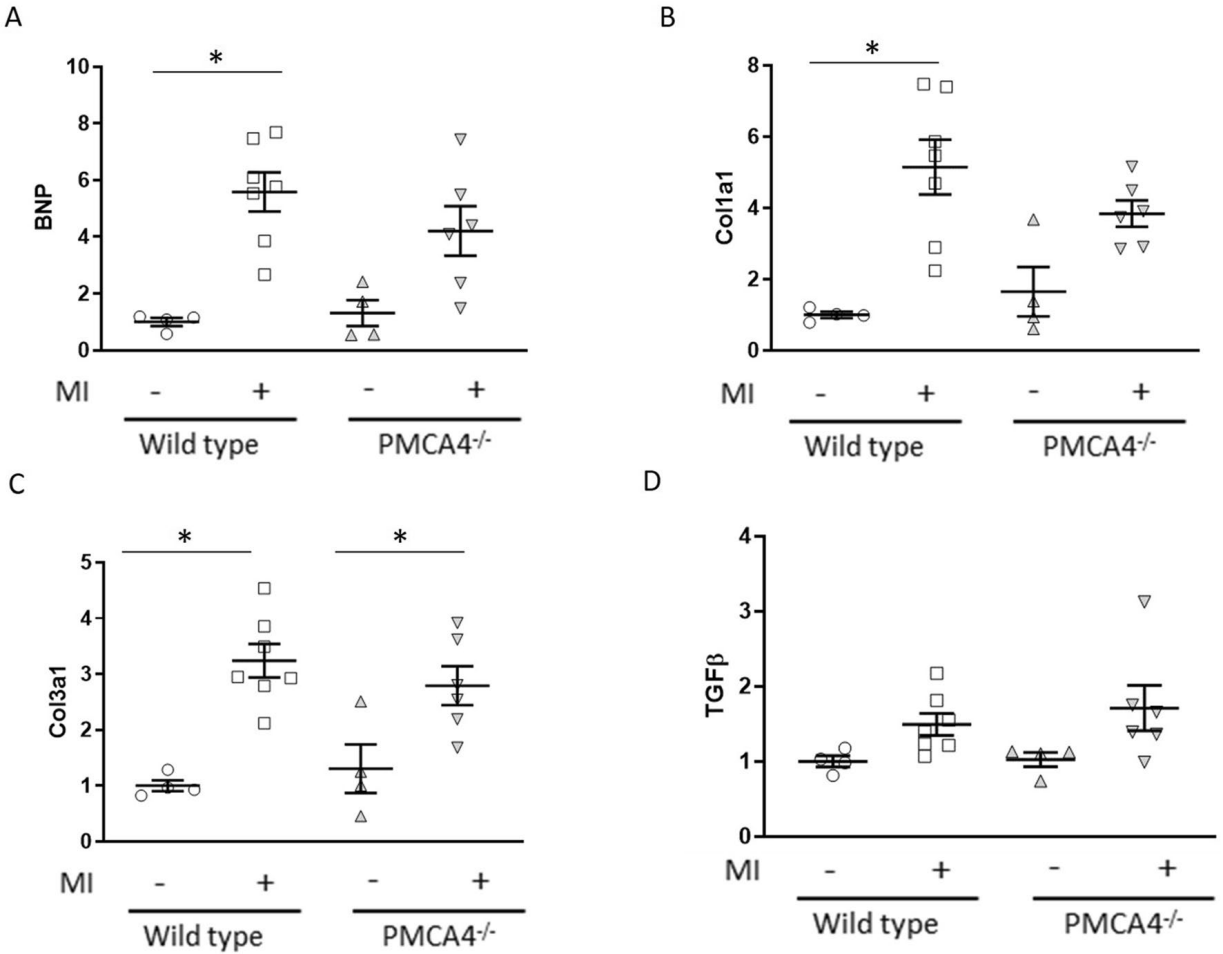
PMCA 4 global deletion does not affect acute remodelling or the fibrotic response post-MI. Myocardial gene expression was examined by qRT-PCR in WT (clear points) and PMCA4^−/−^ (grey points) hearts 2 days post sham or MI surgery for (**A**) Hypertrophic marker BNP, along with markers for fibrosis (**B**) Col1a1, (**C**) Col3a1 and (**D**) TGFβ. Shams n = 4, WT MI n = 7, PMCA4^−/−^ MI n = 6. *p < 0.05.

We then assessed whether hypertrophic remodelling was affected at this early stage post-MI by examining *Bnp* expression. We found this to be increased significantly following MI in WT hearts, and to a lesser extent in *PMCA4*^*−/−*^ hearts compared to shams (Fig. 5A). We found a similar pattern of increased expression of fibrotic markers *Col1a1* and *Col3a1* 2 days post-MI compared to sham controls, but again this did not differ significantly between knockout and WT hearts (Fig. 5B,C). Transforming growth factor β (TGF-β) plays an important role in mediating the switch from inflammation to fibrotic remodelling in the infarcted heart^31^, however we did not find levels to be increased compared to sham in either genotype 2 days after MI surgery (Fig. 5D). Overall this data suggests that the inflammatory and fibrotic responses in the myocardium were comparable amongst WT and *PMCA4*^*−/−*^ mice during the acute phase post-MI.

**Figure 6:**
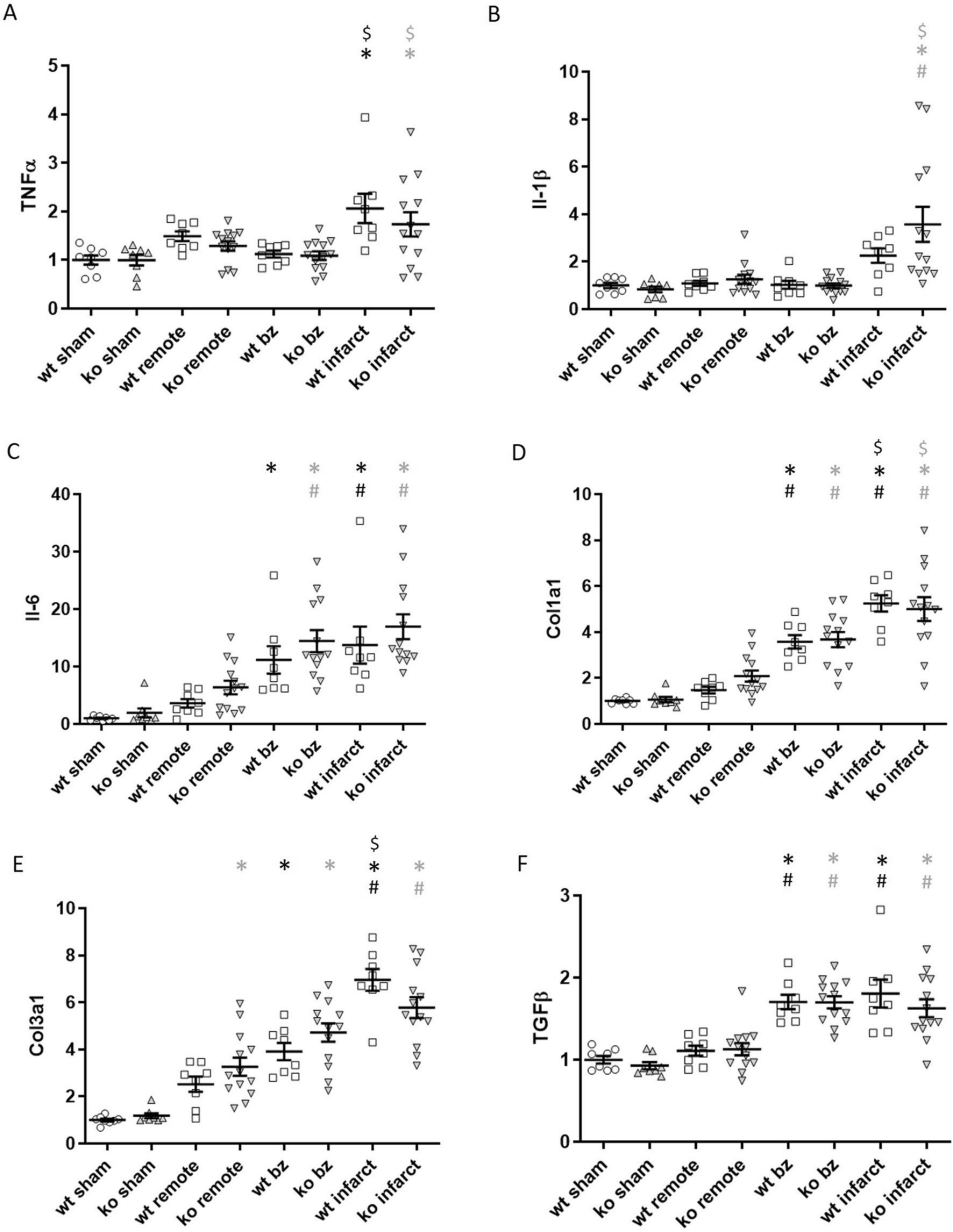
PMCA 4 global deletion does not affect chronic inflammation or fibrosis 6 weeks post MI. Regional gene expression was examined by qRT-PCR in WT (clear points) and PMCA4^−/−^ (grey points) hearts 6 weeks post sham or MI surgery for pro-inflammatory cytokines (**A**) TNFα, (**B**) Interleukin-1β and (**C**) Interleukin-6, in addition to fibrosis markers (**D**) Col1a1, (**E**) Col3a1 and F) TGFβ. bz = infarct border zone. Shams n = 8, WT MI n = 8, PMCA4^−/−^ MI n = 13. *p < 0.05 versus sham, # *p* < 0.05 versus remote, $ *p* < 0.05 versus border zone.

We then went onto examine regional levels of inflammatory and fibrotic markers in heart tissue 6 weeks post-MI. Expression of inflammatory cytokines TNFα and Il-1β was similar in remote and border regions of MI hearts compared to sham hearts, whilst being elevated in the infarcted region (Fig. 6A,B). Il-6 expression was higher in border and infarct regions compared to the remote region and sham hearts (Fig. 6C), however we did not observe any significant differences in inflammatory cytokine expression amongst matched regions of WT and *PMCA4*^*−/−*^ hearts. Il-10 levels were undetectable in regional samples 6 weeks post-MI, presumably due to low expression (data not shown). Expression of fibrosis markers *Col1a1* and *Col3a1* was increased in the border region and to a greater extent the infarct of MI hearts compared to sham and remote regions, whilst *Tgfb* levels were also elevated in border and infarct regions (Fig. 6D,E). No significant differences in inflammatory gene expression were observed between matched regions of WT and *PMCA4*^*−/−*^ hearts 6 weeks post-MI, suggesting a comparable chronic fibrotic response.

### Cardiac fibroblast-specific Pmca4 deletion does not affect outcomes post-MI

Our previous work has suggested that PMCA4 might have distinct functions in different cell types, such as cardiac fibroblasts^20^, cardiomyocytes^12,18,19^ and endothelial cells^22^. Therefore, to study the effects of cell specific deletion of *Pmca4* we first analysed our fibroblast specific PMCA4 knockout mice (*PMCA4*^*fko*^). Previously, we found that *Pmca4* deletion specifically from cardiac fibroblasts protected the myocardium during pressure overload through increased secretion of sFRP2, a factor previously shown to improve outcomes after MI^28^. We therefore performed LAD ligation in our fibroblast-specific PMCA4 knockout mice to investigate whether survival benefits were manifested through deletion from this cell type. We had previously generated this mouse line using *Cre*-recombinase under the control of the promoter for Periostin, and shown deletion of PMCA4 from cardiac fibroblasts without affecting expression in cardiomyocytes^20^.

**Figure 7:**
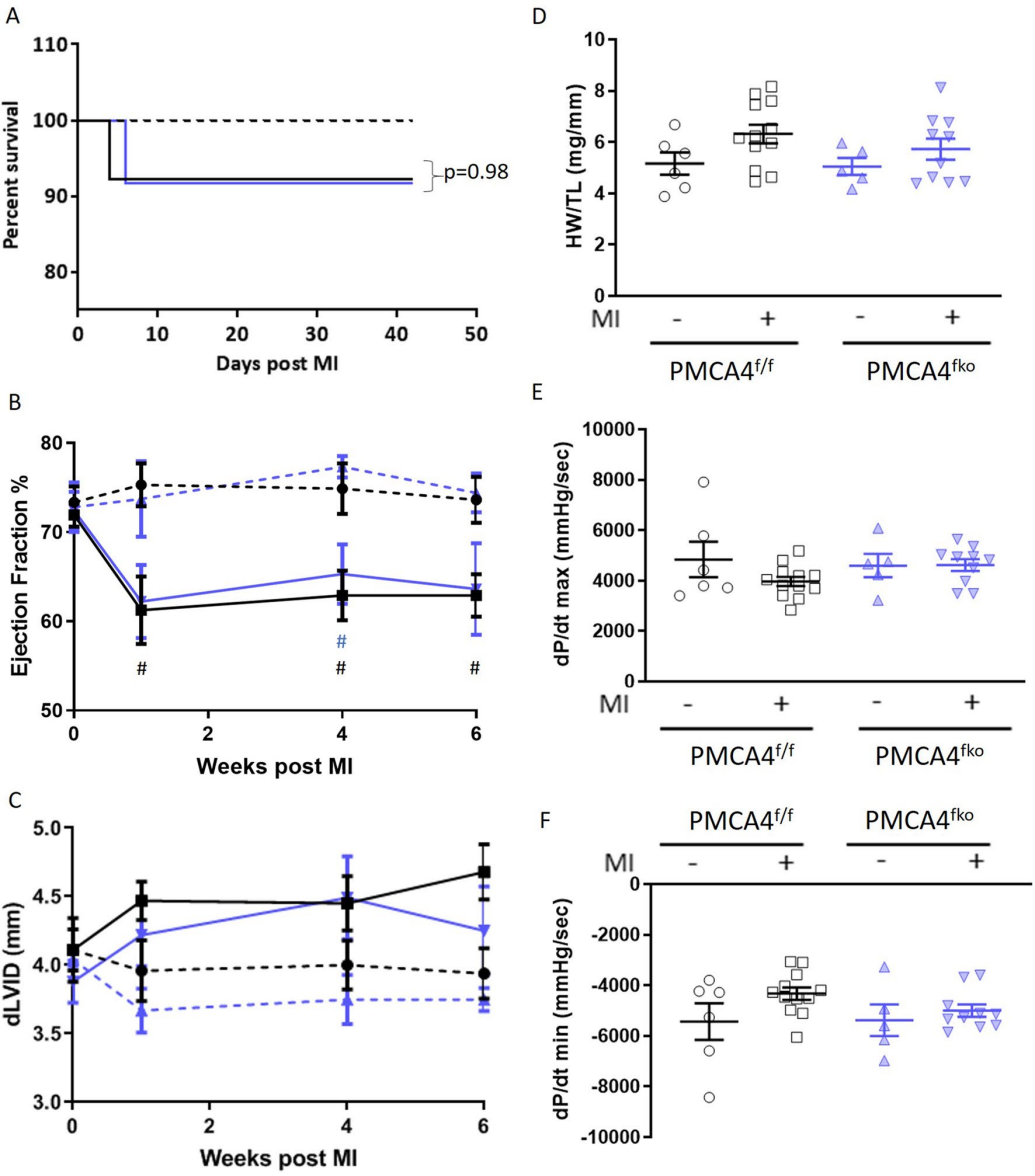
PMCA 4 deletion from fibroblasts does not affect cardiac structure or function post-MI. (**A**) Kaplan–Meier survival plots for PMCA4^flox/flox^ (black) and PMCA4^fko^ mice (blue) followed for 6 weeks post MI (solid lines) or sham (dashed lines) surgery. Starting populations: control sham n = 6, control MI n = 13, PMCA4^fko^ sham n = 6, PMCA4^fko^ MI n = 12. (**B**) Ejection fraction and (**C**) Diastolic left ventricular diameter tracked for 6 weeks post-MI or sham surgery by echocardiography. #*p* < 0.05 versus sham control at matched time point (**D**) Heart weight/tibia length ratio 6 weeks post-MI or sham surgery. (**E**) dP/dt max and (**F**) dP/dt min 6 weeks post MI or sham surgery. control sham n = 6, control MI n = 12, PMCA4^fko^ sham n = 6, PMCA4^fko^ MI n = 11.

Both *PMCA4*^*fko*^ mice and *PMCA4*^*flox/flox*^ controls experienced a 92% survival rate following 6 weeks of MI, with only 1 death per group (Fig. 7A). No deaths were observed in sham mice of either genotype. Cardiac ultrasound revealed that MI mice exhibited a small reduction in ejection fraction 1 week post-MI compared to sham controls, which did not deteriorate further thereafter (Fig. 7B); however ejection fraction was not altered in *PMCA4*^*fko*^ MI mice compared to MI controls at any time point. We did not see any differences in survival, ejection fraction or hypertrophy between genotypes when analysing male and female mice separately (Supplementary Table S3). Neither *PMCA4*^*fko*^ nor *PMCA4*^*flox/flox*^ control hearts showed significant signs of left ventricular dilation following MI (Fig. 7C). Normalised heart weight was not affected in *PMCA4*^*fko*^ mice compared to controls 6 weeks post-MI (Fig. 7D), whilst the force of contraction and relaxation was also comparable amongst groups (Fig. 7E,F). This data suggests that *Pmca4* deletion from cardiac fibroblasts did not affect cardiac function, remodelling or outcomes post-MI.

### Cardiomyocyte-specific deletion of *Pmca4* does not alter the phenotype post-MI

**Figure 8:**
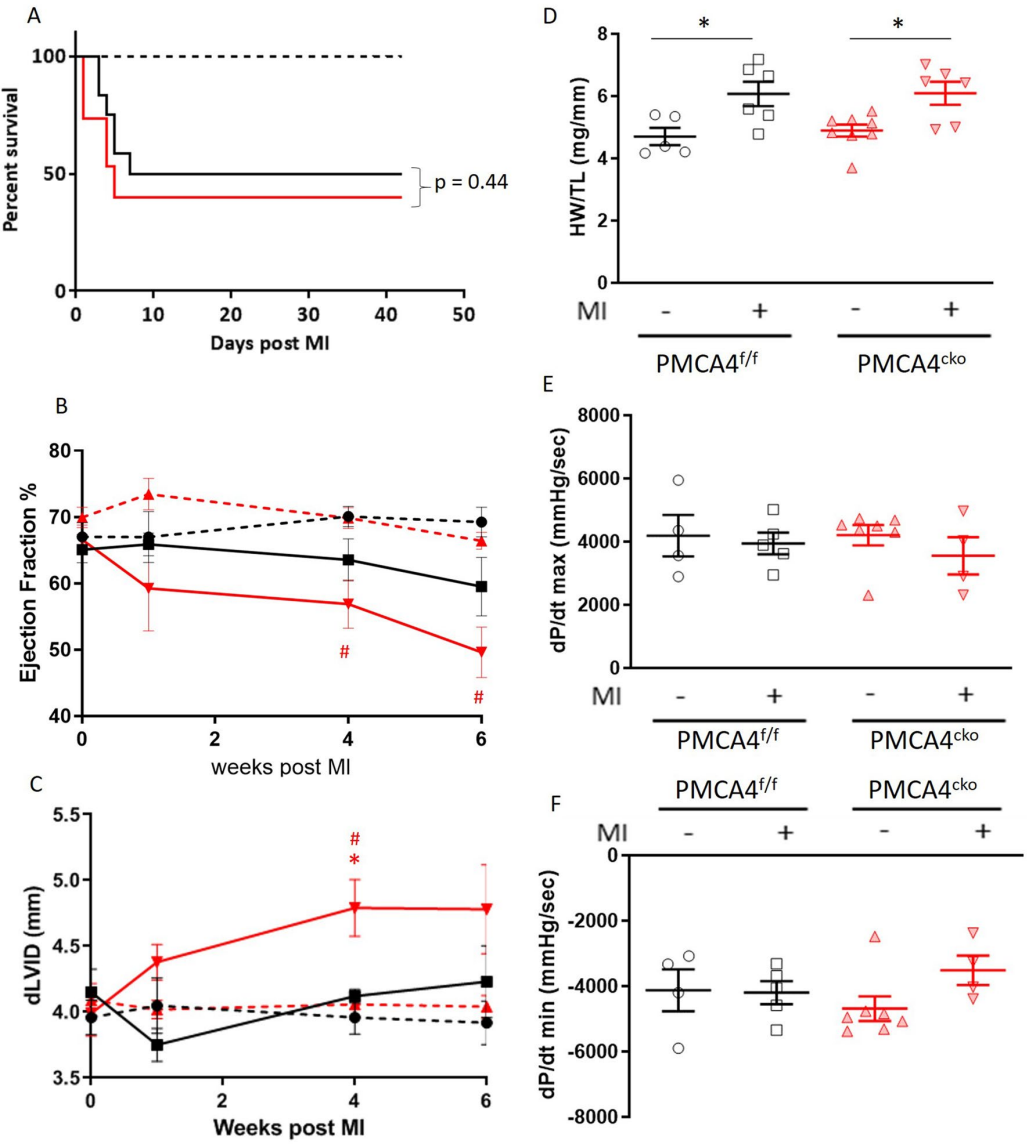
PMCA 4 deletion from cardiomyocytes does not affect cardiac structure or function post-MI. (**A**) Kaplan–Meier survival plots for PMCA4^flox/flox^ (black) and PMCA4^cko^ mice (red) followed for 6 weeks post MI (solid lines) or sham (dashed lines) surgery. Starting populations: control sham n = 5, control MI n = 12, PMCA4^cko^ sham n = 8, PMCA4^cko^ MI n = 15. (**B**) Ejection fraction and (**C**) Diastolic left ventricular diameter tracked for 6 weeks post-MI or sham surgery by echocardiography. # *p* < 0.05 versus sham control at matched time point, * *p* < 0.05 versus control MI at matched time point. (**D**) Heart weight/tibia length ratio 6 weeks post-MI or sham surgery. * *p* < 0.05 (**E**) dP/dt max and (**F**) dP/dt min 6 weeks post MI or sham surgery. control sham n = 5, control MI n = 6, PMCA4^cko^ sham n = 8, PMCA4^cko^ MI n = 6.

Inducible overexpression of *Pmca4* in cardiac myocytes has been shown to improve survival and reduce infarct size following MI^32^. We therefore examined whether cardiomyocyte-specific deletion of *Pmca4* (*PMCA4*^*cko*^ mice) also has an effect on outcomes after LAD ligation. We have previously generated *PMCA4*^*cko*^ mice, and confirmation of the specific ablation of PMCA4 from their cardiomyocytes has been described^20^. *PMCA4*^*cko*^ mice were subjected to MI for 6 weeks. *PMCA4*^*cko*^ mice exhibited a 40% 6 week survival rate, which did not differ significantly from that of their littermate *PMCA4*^*flox/flox*^ controls (Fig. 8A).

*PMCA4*^*cko*^ mice exhibited a small reduction in ejection fraction at 4 and 6 weeks post-MI compared to sham mice, but function did not differ significantly from *PMCA4*^*flox/flox*^ MI controls (Fig. 8B). *PMCA4*^*cko*^ mice also displayed signs of increased left ventricular dilation at 4 weeks compared to controls (Fig. 8C). Both *PMCA4*^*cko*^ and *PMCA4*^*flox/flox*^ control mice displayed evidence of hypertrophy as assessed by heart weight tibia length ratio at the end of the 6 week MI period when compared to their respective sham operated controls (Fig. 8D); however the extent of this did not differ by genotype. When analysing male and female cohorts separately, we again found no significant differences in survival, ejection fraction or hypertrophy amongst *PMCA4*^*cko*^ and *PMCA4*^*flox/flox*^ control MI mice (Supplementary Table S4). Haemodynamic examination of the maximal and minimal forces of contraction and relaxation found no significant differences amongst *PMCA4*^*cko*^ and *PMCA4*^*flox/flox*^ control mice 6 weeks after MI (Fig. 8E,F). Taken together this data suggests that *Pmca4* ablation specifically from cardiomyocytes did not impact survival after MI, and had little impact on cardiac structure and function.

### Pharmacological inhibition of PMCA4 does not impact outcomes after MI

**Figure 9:**
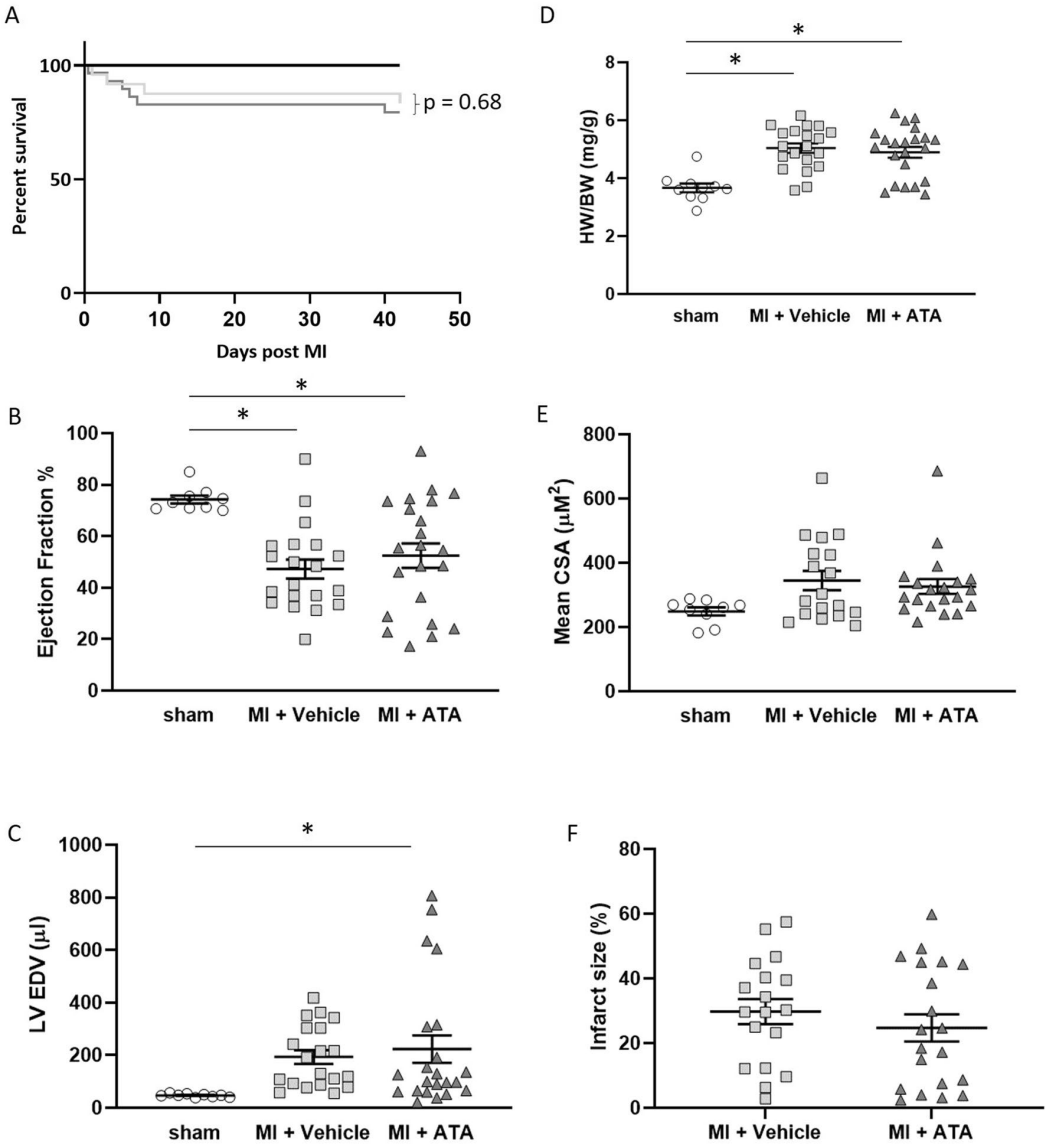
Treatment with PMCA 4 specific inhibitor does not affect cardiac structure or function post-MI. (**A**) Kaplan–Meier survival plots for mice subjected to sham surgery (black), or left anterior descending coronary artery ligation with vehicle (light grey) or PMCA4 inhibitor ATA treatment (dark grey) followed for 6 weeks post MI. Starting populations: sham n = 10, MI + vehicle n = 24, MI + ATA n = 29. (**B**) Left ventricular ejection fraction and (**C**) End diastolic volume 6 weeks post-MI or sham surgery as measured by echocardiography. (**D**) Heart weight/body weight ratio 6 weeks post-MI or sham surgery. (**E**) Mean myocyte cross sectional area 6 weeks post-MI or sham surgery. (**F**) Infarct size 6 weeks post MI. sham n = 10, MI + vehicle n = 20, MI + ATA n = 22 * *p* < 0.05.

To investigate if there is any difference in outcome between genetic and pharmacological inhibition of PMCA4, we performed experiments to assess the effects of pharmacological inhibition of PMCA4 during MI. We have previously identified Aurintricarboxylic acid (ATA) as a potent inhibitor of PMCA4^24^. ATA treatment can mimic the anti-hypertrophic effects of *Pmca4* gene ablation during pressure overload^20^. We therefore performed LAD ligation in mice implanted with osmotic minipumps to deliver 5 mg/kg BW/day of ATA or vehicle control and followed up for a period of 6 weeks. Both MI groups experienced similar survival rates following MI, with 83% of vehicle and 79% of ATA treated mice surviving, respectively (Fig. 9A). Left ventricular ejection fraction as assessed by echocardiography was decreased in both MI groups compared to sham operated controls, but did not differ by treatment (Fig. 9B). Similarly, left ventricular end diastolic volume and normalised heart weight increased comparably in ATA and vehicle treated mice after MI (Fig. 9C,D), whilst no significant differences were found in mean myocyte cross-sectional area amongst groups (Fig. 9E). Furthermore, we found no difference in the infarct size between ATA and vehicle treated mice 6 weeks post-MI (Fig. 9F). Overall this data suggests that specific PMCA4 inhibition does not affect survival, cardiac function or remodelling after MI.

## Discussion

Overall this study shows that *Pmca4* silencing does not affect chronic cardiac remodelling following myocardial infarction, when induced by permanent ligation of the left anterior descending coronary artery. These results were consistent amongst global and tissue-specific knockout models, and confirmed by pharmacological targeting with the specific PMCA4 inhibitor ATA. Global ablation of *Pmca4* did however reduce the occurrence of ectopic beats acutely post-MI, which may be associated with a small but non-significant survival benefit during this period.

It was important to conduct an investigation into the therapeutic potential of using *Pmca4* inhibition to treat remodelling after MI. Our previous work in the pathological setting of pressure overload had shown that *Pmca4* deletion protected the myocardium from the development of cardiac hypertrophy and remodelling^20^. Moreover, deletion was able to preserve long term function and prevent heart failure development, whilst pharmacological inhibition was able to reverse established hypertrophy. These beneficial outcomes were achieved through the paracrine secretion of the Wnt inhibitor sFRP2 from *Pmca4-*silenced fibroblasts, with a subsequent reduction in canonical Wnt signalling^20^.

Wnt signalling plays an essential role in cardiac development, supporting the proliferation of cardiac progenitor cells and controlling their migration and differentiation during heart formation^33^. In the healthy adult heart the Wnt pathway is quiescent; however it is reactivated in response to pathological stress and contributes towards adverse cardiac remodelling^34^, including following myocardial infarction^27,35–38^. Moreover, it has been shown that sFRP2 treatment is capable of reducing infarct size, attenuating cardiomyocyte apoptosis and improving long term cardiac function in experimental MI and hypoxia models^28,39,40^. This was found to be the case both when administered exogenously^28^, and upon its paracrine release from mesenchymal stem cells^39,40^.

It was therefore surprising to find that *PMCA4*^*−/−*^ mice, which have elevated levels of sFRP2 in their cardiac fibroblasts^20^, exhibited similar scar size and cardiac function to controls throughout the 6 week period after MI. This could be due to endogenous upregulation of sFRP2 in WT fibroblasts, which has been noted to occur during the first week after MI^35^. Indeed, we found sFRP2 levels to be dramatically increased in both control and *PMCA4*^*−/−*^ tissue at the whole heart level 6 weeks post-MI, particularly in the infarct and border regions. Analysis of β-catenin target gene *Axin2* expression suggested a possible reduction of *Wnt* signalling in infarcts of *PMCA4*^*−/−*^ mice, but this didn’t translate to any observable physiological effects. It should also be noted that sFRP2-null mice, and hamsters treated with antibody-mediated sFRP2 blockade, have been shown to have protection from fibrosis and improved cardiac function after MI^41,42^, indicating that sFRP2’s role in the infarcted heart may be multi-faceted, and more complex than in the TAC heart. Furthermore, Wnt signalling has been shown to be highly active in a number of other cell types in the infarcted myocardium including in progenitor, endothelial cell and leucocyte populations^37,38^. Therefore, it is also possible that regulating sFRP2 signalling in cardiac fibroblasts is not sufficient to affect Wnt activity at the organ level.

The differences in phenotype between *Pmca4*-null hearts after TAC and MI also likely reflects significant distinctions between the pathological processes that drive concentric hypertrophic remodelling following pressure overload, compared to eccentric remodelling that occurs in volume overloaded MI hearts^43,44^. Pressure and volume overload trigger different biomechanical and neurohumoral pathways, with a far greater extent of protein synthesis occurring in pressure overloaded TAC hearts^43^. In contrast, post-MI remodelling is largely driven by an extensive inflammatory response to widespread necrosis, aimed at repairing the damaged myocardium^45^. This involves the recruitment of large numbers of immune cells to clear debris from the infarct^46^, and fibroblasts, whose proliferation and transformation into myofibroblasts leads to reparative fibrosis and scar maturation^47^. Ultimately, infarct size and healing are thought to be the major determinants of adverse remodelling after MI^47^. In TAC hearts, while there is evidence of a macrophage-led inflammatory response, this is more transient and to a lesser extent than after MI^48^, and is not associated with vast fibroblast recruitment.

The rationale to investigate the role of *Pmca4* inhibition after MI went beyond its regulation of Wnt signalling in cardiac fibroblasts. PMCA4 has been shown to play an important role in mediating angiogenesis through its actions in endothelial cells, with genetic ablation and pharmacological inhibition each enhancing cell migration and improving reperfusion following hindlimb ischaemia^22,26^. These studies also contributed towards our hypothesis of beneficial outcomes post-MI, given that stimulation of angiogenesis in the infarcted heart has been shown to improve chronic left ventricular remodelling and function^49^. Although we did not measure vessel formation in this study, we did not see any evidence of improved chronic outcomes. It may be that PMCA4’s regulation of angiogenesis after MI would be better studied in a model of ischaemia with reperfusion rather than following permanent coronary artery ligation, but this was beyond the scope of the current study.

Despite our findings in this study, it is likely that PMCA4 does play a role in regulating the response to ischaemic injury in the heart. A recent study has found that transgenic mice overexpressing human PMCA4b specifically in cardiomyocytes displayed less cardiac hypertrophy and improved fractional shortening following LAD ligation^32^. This was the case both when expressed from birth, or inducibly 4 weeks after MI, and was associated with a reduction in infarct size and improved survival^32^. The authors found PMCA4′s regulation of nNOS mediated nitric oxide signalling to be responsible for these protective effects^32^. In the present study, we found that specific deletion of *Pmca4* from cardiomyocytes did not have a significant effect on outcomes following MI. This was also the case in our previous study during pressure overload, in which cardiac hypertrophy was not affected when PMCA4 levels were reduced only in cardiomyocytes^20^. This is likely due to differences in the mechanism of nNOS regulation between PMCA4 overexpression and knockout models. We have found that increased expression of PMCA4 inhibits nNOS activity through reducing local calcium^18,19^, whereas nNOS is delocalised from the membrane compartment in *PMCA4*^*−/−*^ hearts^12^.

This does not rule out an effect of *Pmca4* silencing in other cell types involved in the response to MI. We did note significantly fewer instances of premature atrial/junctional contractions upon ECG examination on the day following LAD ligation in *PMCA4*^*−/−*^ mice compared to controls, which to some extent may have protected against sudden death during the first 72 h post-MI. Indeed, premature atrial complexes are associated with increased mortality from sudden cardiac death^50^; however telemetric ECG recordings would need to be conducted in order to verify whether lethal arrhythmias were the cause of excess deaths amongst WT mice in our experiments.

The acute response to infarction begins with an immune response to necrotic signals, sparked by the release of inflammatory cytokines and chemokines^45^. Infiltration of leucocytes and their adherence to endothelial cells amplifies the inflammatory response and allows for clearance of necrotic cells^45^; however, overactive inflammation during the early stages after MI can cause electrophysiological remodelling, arrhythmia and sudden death^29^. In addition, suppression of inflammation is important in order to proceed to the reparative phase of infarct healing, which involves fibroblast proliferation, extracellular matrix secretion and eventually fibrotic scar maturation^45^. In the present study we did not find *Pmca4* global ablation to modulate myocardial inflammatory or fibrotic gene expression at either 2 days or 6 weeks post-MI, which may suggest that regulation of arrhythmia is more likely to contribute to the phenotype than inflammation and fibrosis. However we would not completely rule out a role in the remodelling process, as it is possible that PMCA4 could regulate processes at another time point, for example the transition to the reparative and maturation phases of infarct healing. Furthermore, in order to fully rule out any involvement in inflammation, it would be pertinent to investigate the immune cell response in our mice.

Studies of the immune cell response following MI in mice have shown that after early neutrophil infiltration on day 1 post-MI, the macrophage population predominates by day 3, and in particular M1 inflammatory macrophages^46^. Thereafter, M2 macrophages, monocyte and T-cell populations act to supress inflammation^45,46^. Therefore it could be that PMCA4 regulates inflammatory processes following MI in one or more cell types not examined during our study, such as endothelial cells, macrophages, or T-cells, which may account for the slight improvement in survival and protect from the development of acute arrhythmic events in our *Pmca4* global knockout mice. Indeed, PMCA4 has been previously shown to play an important role during T-cell activation^51,52^. However, given the minimal effect of global genetic and pharmacological *Pmca4* inhibition on overall outcomes post-MI, it would be beyond the scope of this study to produce further tissue-specific knockouts to investigate such a role.

We also examined whether sex played a role in determining post-MI outcomes in each knockout strain in our study. This suggested that improved survival in mice with global *Pmca4* deletion may be restricted to female mice, which may account for comparable survival rates amongst vehicle and PMCA4 inhibitor-treated mice as these experiments were performed exclusively in males. However, it should be noted that when performing our 2 day MI experiments we observed deaths in female PMCA4^−/−^ mice at a rate comparable to WT, and so further investigation would be required in order to confirm a sex-dependent survival benefit.

An additional point of interest in our study is the variation in survival rate between WT mice and targeted flox strains, which ranges from 92% in *PMCA4*^*fko*^-floxed controls, to 50% in *PMCA4*^*cko*^-floxed controls and WT strains. It is likely that the different responses are due to background strain, which has been shown to play a significant role in the response to MI in terms of survival, cardiac rupture, dilatation and function. A study comparing 5 different mouse strains found survival rates after MI to vary greatly by strain, ranging from 33% in 129S6 mice to 85% in BalbC mice^53^. Each of the 3 lines used in our study are bred on different mixed backgrounds, including C57Bl/6J for *PMCA4*^*fko*^ mice^54^ and ICR for *PMCA4*^*cko*^ mice^55^, which likely accounts for the response to MI in each strain.

Overall these results demonstrate that PMCA4 ablation and inhibition do not significantly affect chronic outcomes in a model of permanent myocardial infarction, but may reduce the susceptibility to acute arrhythmia and sudden death. In addition, PMCA4 inhibition presents a promising target in a number of other cardiovascular areas including pressure overload induced cardiac hypertrophy^20^, the promotion of angiogenesis^22,26^ and the reduction of blood pressure^25^. Further studies looking to develop PMCA4 inhibitors as a translational therapy may want to focus on these processes, along with alternative models such as ischaemia with reperfusion.

## Methods

### Animals

*PMCA4*^*−/−*^ mice with targeted deletion of exons 2–3 of the *Pmca4* gene used in this study were previously generated^23^. *PMCA4*^*flox/flox*^ mice, cardiomyocyte-specific *PMCA4*^*cko*^ and fibroblast-specific *PMCA4*^*fko*^ mice were also previously generated in our laboratory, with Cre-mediated excision of exons 2–3 of the *Pmca4* gene driven by the *αMHC* and *Periostin* promoters in cardiomyocytes and fibroblasts, respectively^20^. 8–12 week old mice of both sexes were used in each strain, with subjects age and sex matched between knockouts and littermate controls. 8 week old male C57BL/6 mice weighing 20–23 g were used for ATA experiments.

Animals were housed in a specific-pathogen free environment and studies were performed in accordance with the United Kingdom Animals (Scientific Procedures) Act 1986. All studies were approved by the University of Manchester Ethics Committee. Researchers were blinded to genotype and treatment during data collection and analysis. All animal experiments were conductance in accordance with the ARRIVE guidelines^56^. This study does not have any implications on replacement, refinement and reduction.

### Coronary artery ligation

Myocardial infarction was induced by permanent ligation of the left anterior descending (LAD) coronary artery. Mice received pre-operative analgesia via subcutaneous injection of buprenorphine (0.1 mg/kg body weight). They were induced and intubated under 5% isofluorane, and thereafter mechanically ventilated (200 breaths per minute, tidal volume 0.1 ml) and maintained in a stable plane of anaesthesia with 3% isofluorane in 100% O_2_. A left-sided thoracotomy was performed and the ribs retracted to expose the heart. The LAD artery was ligated above its bifurcation using *#*8.0 suture, approximately 1 mm below the left atrial appendage. Visual confirmation of ischaemia was observed by a paling of the ventricular tissue distal to the ligation. In sham operations, a suture was passed through the myocardium but withdrawn without ligation. The thorax and chest were then sutured shut, and mice allowed to recover at 30 °C before return to normal housing.

### ATA minipump experiments

Immediately following sham or coronary artery ligation surgery, osmotic minipumps (Alzet) containing either ATA or vehicle (50% DMSO, 50% water) were implanted subcutaneously. A small skin incision was made dorsally to create a pocket into which the minipump was implanted, following which the skin was sutured closed. ATA mice received a dose of 5 mg/kg BW/day for a period of 6 weeks.

### Echocardiography

To evaluate cardiac function during 6 weeks follow up to MI induction, transthoracic echocardiography was performed using a Visualsonics Vevo 770 imaging system fitted with an RMV-707B scanhead. Mice were anaesthetised using 1.5% isofluorane, and images were acquired in parasternal long and short axis views to measure wall thickness and chamber dimensions, from which ejection fraction was calculated. Researchers were blinded to mouse genotype and treatment during the procedure. Measurements were obtained using the leading-edge method over a minimum period of three cardiac cycles.

### Haemodynamics

Analysis of haemodynamic function was performed via invasive catheterization of the left ventricle under terminal anaesthesia (250 mg/kg body weight tribromoethanol injected i.p.). Mice were placed in a supine position and a midline cervical incision made to expose the right carotid artery. This was tied at its bifurcation and occluded proximally to allow an incision to be made, through which a 1.4F pressure–volume catheter (SPR-839, Millar Instruments) was inserted, and fed down the ascending aorta into the left ventricle. Recordings were collected using a PowerLab system once traces had stabilised. Inotropic and lusitropic function were assessed by the maximal and minimal rates of pressure change, dP/dt_max_ and dP/dt_min_, respectively.

### Electrocardiogram

3-lead ECG recordings were collected 24 h post-MI under anaesthesia with 1.5% isofluorane. Mice were placed on a heat pad set to 37 °C and electrodes inserted into the muscle of the right and left forelimb, and right hindlimb. ECG traces were recorded for a period of 15 min on a PowerLab system, and examined for the occurrence of ectopic beats during this time using LabChart software (AD instruments).

### Tissue collection

Mice were euthanised under terminal anaesthesia by injection of 1 M KCl solution directly into the LV chamber to arrest the heart in diastole. Hearts were then flushed of blood with PBS. For 2 day experiments hearts were collected onto dry ice for subsequent analysis of area at risk by triphenyltetrazolium chloride (TTC) stain. For 6 week experiments, hearts were sliced into transverse sections 1 mm in thickness using a mouse heart slicing matrix (Harvard Apparatus). Sections were embedded in OCT compound and frozen using a bath of 2-methylbutane and dry ice, following which they were stored at − 80 °C.

### Infarct size measurement

Infarct size was determined following Masson’s trichrome staining of transverse sections using the midline length measurement technique as described by Takagawa et al.^57^. 1 mm thick OCT embedded slices taken at 5 levels of the ventricle were sectioned on a cryostat at a thickness of 6 µm and mounted onto glass slides. After equilibrating to room temperature, sections were fixed in 4% PFA for 15 min and then stained with Masson’s trichrome using a standard protocol. Slides were imaged on a 3D Histech Panoramic slide scanner in the University of Manchester bioimaging facility, and infarct size was measured using Image J software. The LV midline circumference was determined for each section by drawing a line equidistant to the epicardium and endocardium. The infarct length in each section was determined by drawing a midline through the infarct, where the scar constituted > 50% of the myocardial wall thickness. Infarct size as a percentage of the left ventricle was calculated as the sum of the infarct lengths at each level divided by the sum of the total LV circumferences at each level, × 100.

### RNA and qPCR

Following cryosectioning, the remaining heart tissue was retrieved from the OCT, divided into infarct, border and remote regions and placed in RNA later solution (Qiagen) for storage as per manufacturer instructions. Sham tissue was also OCT embedded, frozen and retrieved into RNA later. For RNA extraction, tissue was manually dissociated in a dounce homogenizer using Trizol reagent (Thermo), and then processed according to the manufacturers’ instructions. RNA concentration was determined using a Nanodrop 8000 spectrophotometer, following which 1 µg total RNA was reverse transcribed to cDNA using a high capacity cDNA reverse transcription kit (Thermo). qPCR was conducted on a 7500 Fast RT-PCR system (Applied Biosystems) using Brilliant III Ultrafast SYBR green (Agilent) and Quanitect Primer Assays (Qiagen). Samples were loaded in triplicate and gene expression was determined using the 2^−ΔΔCT^ method^58^, using GAPDH and β-actin as endogenous controls.

### Area at risk measurement by TTC staining

Hearts were stored on dry ice to keep tissue metabolically active. They were then sectioned into 1 mm transverse slices at 5 levels through the ventricle using a mouse heart slicing matrix. Sections were incubated in 0.5% TTC solution in pH7.4 buffered PBS for 15 min at 37 °C. Sections were then washed in PBS and immediately imaged using a stereomicroscope. Area at risk was determined using Image J software, by measuring the sum of the white (infarcted) area at each level and dividing by the sum of the total LV area at the 5 levels, multiplied by 100.

### Data analysis and statistics

Data was analysed using Microsoft Excel and GraphPad Prism software, and are presented as mean ± SEM. Data was analyzed using one- or two-way ANOVA followed by Bonferroni post hoc tests where appropriate, and comparisons between two groups were performed using Student’s t-test. Survival analysis was performed using the Log-rank (Mantel-Cox) test. Fisher’s exact test was used to calculate contingency of arrhythmia. *p*-values < 0.05 were considered statistically significant.

## Supplementary Information

Supplementary Information.
