# Supplementary material for: PMCA4 inhibition does not affect cardiac remodelling following myocardial infarction, but may reduce susceptibility to arrhythmia
Source: Sci Rep. 2021 Jan 15;11:1518. doi: 10.1038/s41598-021-81170-2 (PMC7810749; doi:10.1038/s41598-021-81170-2)
Supplement: Supplementary file 1 — Supplementary Information. [file 41598_2021_81170_MOESM1_ESM.pdf]

Supporting information

PMCA4 inhibition does not affect cardiac remodelling following myocardial infarction, but may reduce susceptibility to arrhythmia

Nicholas Stafford, Min Zi, Florence Baudoin, Tamer M.A. Mohamed, Sukhpal Prehar, Elizabeth J. Cartwright, Roberto Latini, Ludwig Neyses, Delvac Oceandy

Supplementary Figure S1

Supplementary Tables S1-4

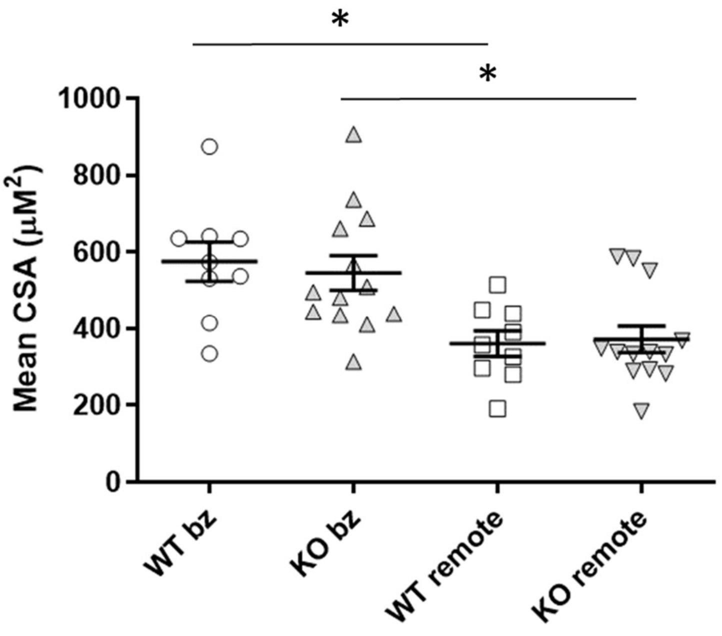

Supplementary figure S1. Cardiomyocyte cross sectional area in PMCA4$^{-/-}$ mice post-MI

Myocyte cross-sectional area measured in the infarct border region (bz) and remote region of the myocardium in wild type (clear points) and PMCA4$^{-/-}$ mice (grey points). N=9 mice for WT, 13 mice for PMCA4$^{-/-}$, *P<0.05

Supplementary Table S1. Response to 6 week MI in male and female PMCA4$^{-/-}$ mice

Males – 6 week MI cohort				
	WT sham	WT MI	PMCA4$^{-/-}$ sham	PMCA4$^{-/-}$ MI
Total mice	4	10	4	10
Deaths	0	5	0	4
6 week survival (%)	100	50	100	60
6 week EF (%)	70.7 ± 2.8	54.4 ± 6.5	62.9 ± 4.6	59.9 ± 4.5
HW/TL (mg/mm)	4.8 ± 0.2	6.0 ± 0.4	4.7 ± 0.2	5.7 ± 0.3
6 week IS (%)	-	27.6 ± 7.2	-	33.5 ± 3.1
**Females – 6 week MI cohort**				
	WT sham	WT MI	PMCA4$^{-/-}$ sham	PMCA4$^{-/-}$ MI
Total mice	4	8	4	9
Deaths	0	4	0	0
6 week survival (%)	100	50	100	100 ‡
6 week EF (%)	74.0 ± 3.0	52.1 ± 8.1 *	74.2 ± 3.7	55.4 ± 2.7 *
HW/TL (mg/mm)	4.6 ± 0.2	6.0 ± 0.9	4.2 ± 0.2	5.8 ± 0.3
6 week IS (%)	-	28.9 ± 8.7	-	30.4 ± 3.0

Survival, ejection fraction (EF), heart weight normalised to tibia length (HW/TL) and infarct size (IS) data in wildtype and PMCA4$^{-/-}$ mice 6 weeks after sham or MI surgery, grouped by sex . *p<0.05 vs sham, ‡p<0.05 vs WT MI

Supplementary Table S2. Response to 2 day MI in male and female PMCA4$^{-/-}$ mice

Males – 2 day MI cohort				
	WT sham	WT MI	PMCA4$^{-/-}$ sham	PMCA4$^{-/-}$ MI
Total mice	5	10	5	9
Deaths	0	3	0	1
2 day survival (%)	100	70	100	89
2 day AAR (%)	-	30.5 ± 2.3	-	39.3 ± 2.1
Day 1 arrhythmia (%)	0	37.5	0	0
**Females – 2 day MI cohort**				
	WT sham	WT MI	PMCA4$^{-/-}$ sham	PMCA4$^{-/-}$ MI
Total mice	5	14	5	11
Deaths	0	2	0	2
2 day survival (%)	100	86	100	82
2 day AAR (%)	-	35.4 ± 7.0	-	35.8 ± 4.7
Day 1 arrhythmia (%)	0	70	0	37.5

Survival, area at risk (AAR) and arrhythmia data in wildtype and PMCA4$^{-/-}$ mice in 2 day MI experiments, grouped by sex

Supplementary Table S3. Response to 6 week MI in male and female PMCA4^{fko} mice

**Males – 6 week MI**				
	PMCA4$^{f/f}$ sham	PMCA4$^{f/f}$ MI	PMCA4^{fko} sham	PMCA4^{fko} MI
Total mice	3	8	3	6
Deaths	0	1	0	1
6 week survival (%)	100	87.5	100	83.3
6 week EF (%)	71.0 ± 2.8	60.3 ± 2.7	72.5 ± 2.6	53.2 ± 8.5
HW/TL (mg/mm)	6.0 ± 0.3	7.0 ± 0.4	6.1 ± 0.3	6.9 ± 0.3
**Females – 6 week MI**				
	PMCA4$^{f/f}$ sham	PMCA4$^{f/f}$ MI	PMCA4^{fko} sham	PMCA4^{fko} MI
Total mice	3	5	3	6
Deaths	0	0	0	0
6 week survival (%)	100	100	100	100
6 week EF (%)	76.2 ± 4.4	66.6 ± 4.0	76.3 ± 3.7	72.3 ± 3.9
HW/TL (mg/mm)	4.3 ± 0.3	5.4 ± 0.4	4.5 ± 0.2	4.9 ± 0.3

Survival, ejection fraction (EF) and heart weight normalised to tibia length (HW/TL) data in PMCA4$^{flox/flox}$ and PMCA4^{fko} mice 6 weeks after sham or MI surgery, grouped by sex .

Supplementary Table S4. Response to 6 week MI in male and female PMCA4^{cko} mice

Males – 6 week MI				
	PMCA4$^{f/f}$ sham	PMCA4$^{f/f}$ MI	PMCA4^{cko} sham	PMCA4^{cko} MI
Total mice	3	8	4	8
Deaths	0	5	0	6
6 week survival (%)	100	37.5	100	25
6 week EF (%)	67.3 ± 2.2	47.4 ± 2.7 *	67.8 ± 1.6	45.3 ± 3.1 *
HW/TL (mg/mm)	5.1 ± 0.3	7.0 ± 0.2 *	5.2 ± 0.2	6.9 ± 0.2 *
**Females – 6 week MI**				
	PMCA4$^{f/f}$ sham	PMCA4$^{f/f}$ MI	PMCA4^{cko} sham	PMCA4^{cko} MI
Total mice	2	4	4	7
Deaths	0	1	0	3
6 week survival (%)	100	75	100	57.1
6 week EF (%)	72.3 ± 4.5	65.6 ± 3.2	65.2 ± 1.7	51.8 ± 5.4
HW/TL (mg/mm)	4.2 ± 0.0	5.6 ± 0.4	4.6 ± 0.3	5.7 ± 0.4

Survival, ejection fraction (EF) and heart weight normalised to tibia length (HW/TL) data in PMCA4$^{flox/flox}$ and PMCA4^{cko} mice 6 weeks after sham or MI surgery, grouped by sex. *$p<0.05$ vs sham
